# Substance P Is a Mechanoresponsive, Autocrine Regulator of Human Tenocyte Proliferation

**DOI:** 10.1371/journal.pone.0027209

**Published:** 2011-11-01

**Authors:** Ludvig J. Backman, Gloria Fong, Gustav Andersson, Alexander Scott, Patrik Danielson

**Affiliations:** 1 Department of Integrative Medical Biology, Anatomy, Umeå University, Umeå, Sweden; 2 Department of Surgical and Perioperative Sciences, Sports Medicine, Umeå University, Umeå, Sweden; 3 Department of Physical Therapy, University of British Columbia, Vancouver, British Columbia, Canada; 4 Centre for Hip Health and Mobility, Vancouver Coastal Health and Research Institute, Vancouver, British Columbia, Canada; University of Medicine and Dentistry of New Jersey, United States of America

## Abstract

It has been hypothesised that substance P (SP) may be produced by primary fibroblastic tendon cells (tenocytes), and that this production, together with the widespread distribution of the neurokinin-1 receptor (NK-1 R) in tendon tissue, could play an important role in the development of tendinopathy, a condition of chronic tendon pain and thickening. The aim of this study was to examine the possibility of endogenous SP production and the expression of NK-1 R by human tenocytes. Because tendinopathy is related to overload, and because the predominant tissue pathology (tendinosis) underlying early tendinopathy is characterized by tenocyte hypercellularity, the production of SP in response to loading/strain and the effects of exogenously administered SP on tenocyte proliferation were also studied. A cell culture model of primary human tendon cells was used. The vast majority of tendon cells were immunopositive for the tenocyte/fibroblast markers tenomodulin and vimentin, and immunocytochemical counterstaining revealed that positive immunoreactions for SP and NK-1 R were seen in a majority of these cells. Gene expression analyses showed that mechanical loading (strain) of tendon cell cultures using the FlexCell© technique significantly increased the mRNA levels of SP, whereas the expression of NK-1 R mRNA decreased in loaded as compared to unloaded tendon cells. Reduced NK-1 R protein was also observed, using Western blot, after exogenously administered SP at a concentration of 10^−7^ M. SP exposure furthermore resulted in increased cell metabolism, increased cell viability, and increased cell proliferation, all of which were found to be specifically mediated via the NK-1 R; this in turn involving a common mitogenic cell signalling pathway, namely phosphorylation of ERK1/2. This study indicates that SP, produced by tenocytes in response to mechanical loading, may regulate proliferation through an autocrine loop involving the NK-1 R.

## Introduction

Despite recent scientific advances, the mechanisms of chronic tendon pain and thickening, *tendinopathy*, are yet not fully understood. It is now widely accepted that the underlying tissue changes of tendinopathy, commonly thought to be induced by mechanical overload, constitute a non-inflammatory pathology (*tendinosis)* characterized by hypercellularity and capillary proliferation [1], but the pathogenesis remains unclear.

In the last decade, evidence in favour of a new theory of tendinosis pathophysiology has been presented; this hypothesis suggests that signal substances traditionally thought to be confined to neurons are produced by the tendon tissue itself and are involved in the development of tendinosis [2], [3]. The neurochemical mediators produced by primary fibroblastic tendon cells (*tenocytes*) in human tendinosis tendons *in vivo* include acetylcholine [4], [5], [6], catecholamines [7], [8], [9], glutamate [10], and the neuropeptide substance P (SP) [11]. SP, primarily known for its involvement in afferent pain mechanisms, also has efferent effects which may play a role in tendon pathology, such as stimulation of angiogenesis [12]. It has furthermore been found that exogenously administered SP promotes cellular proliferation in tendon wound healing in rats [13], [14]. The immunoreactivity for nerve-related SP is increased in a rat model of Achilles tendon overuse [15], and human Achilles tendinosis is associated with sprouting of SP positive nerve fibres [16].

In our recent studies of human Achilles tendon tissue, we showed that tenocytes express the mRNA for SP (TAC1) [11], and that the preferred SP receptor (neurokinin-1 receptor [NK-1 R]) is widely distributed in human tendons (including on the tenocytes themselves), with more marked expression in tendinosis tissue [11], [17]. NK-1 R belongs to the tachykinin receptor sub-family of G-protein coupled receptors (GPCRs) [18], and it is well established that GPCRs are involved in transducing extracellular signals leading to proliferation [19]. One such well-known pathway following GPCR stimulation is the activation of mitogen-activated protein kinases (MAPKs) [20], [21], such as the extracellular-signal-regulated kinases 1 and 2 (ERK1/2).

Mechanical strain is considered to be an important component in tendon cell activation [22]. However, the possible connection between strain and production of biochemical mediators, such as SP, remains unclear. Nevertheless, it is well known that after prolonged exercise tendons change their biological and biochemical characteristics [22]. For chondrocytes it has been shown that mechanical strain *in vitro* generates an intracellular signalling leading to secretion of SP [23]. Since tendon cells can detect and communicate mechanical signals to the neighbouring cells by the exchange of molecules [23] the understanding of adaptation following strain is probably important in solving the puzzle of tendinosis.

In view of the above, we hypothesise that SP, possibly produced by tenocytes in response to mechanical strain, has a role in tendinosis development, particularly with regard to the induction and/or exacerbation of excessive or uncontrolled tenocyte proliferation, a prominent feature of tendinosis pathology [1].

For this hypothesis to be tested, it is imperative to disclose 1.) whether the SP protein is produced by human tenocytes, 2.) whether the SP production is increased in response to mechanical loading (i.e. strain), 3.) whether SP induces proliferation of human tenocytes, and if so 4.) if this is a specific effect mediated through the NK-1 R, and 5.) if there is evidence of involvement of a common mitogenic cell signalling pathway (activation of MAPKs).

To study these hypothesised effects of SP, good model systems are required. We have recently, in an animal model (rabbit) of Achilles tendinosis [24], [25], experimentally demonstrated that the intratendinous production of SP *in vivo* indeed increases with mechanical loading and that this elevation precedes the tendinosis tissue changes, including hypercellularity [26]. We furthermore showed that exogenously administered SP accelerates tenocyte proliferation, as well as angiogenesis, in the rabbit Achilles tendon during development of tendinosis [27]. Nevertheless, whether these findings may be generalized to human tendon remains unknown; moreover, animal models can pose ethical problems in addition to producing results not directly transferable to human conditions. Therefore, in this study we established a human tendon cell culture model to study the expression of SP and the SP receptor NK-1 R, the endogenous production of SP, with and without mechanical loading, and the effect on the tendon cells *in vitro* by exogenously administered SP, and the mechanistic aspects of such effects.

## Methods

### Ethics statement

The studies were approved by the Regional Ethical Review Board in Umeå and were performed according to the principles of the Declaration of Helsinki. Written informed consent was received from all participants.

### Primary culture of human Achilles tendon cells

**Figure 1:**
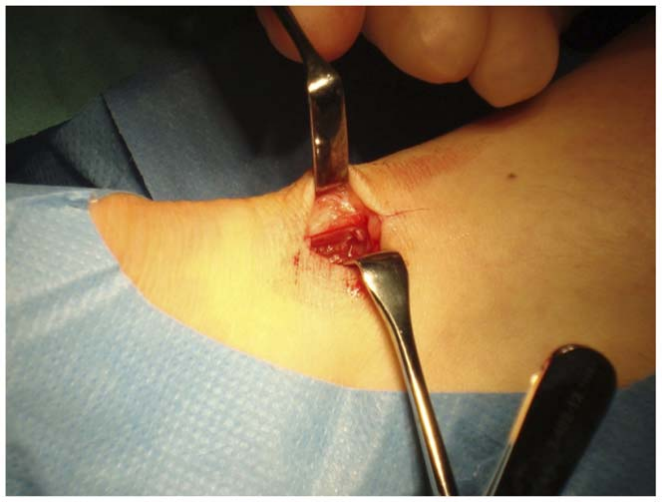
Tissue harvesting. Surgical harvesting of tendon tissue from healthy donors under sterile conditions.

Samples of human Achilles tendons were obtained from the midportion Achilles tendon of healthy donors, defined as voluntary individuals having no history of Achilles tendon pain and demonstrating no structural changes on Colour Doppler ultrasound examination. The donors were all sports active on a recreational level. The ventral side of the tendon was exposed 3–4 cm from its calcaneal insertion through a small lateral skin incision, and the biopsy was taken with a sterile razor blade (Fig. 1). There were no complications as a result of the biopsy procedure.

Samples were washed with sterile Hanks' Balanced Salt Solution (HBSS; Invitrogen; 14170) and carefully dissected and minced using a sterile razor blade before they were enzymatically digested at 37°C, using collagenase (Clostridopeptidase A, C-0130 Sigma) diluted in D-MEM (Invitrogen; 11960) to obtain a concentration of 2 mg/ml. The digestion product was then centrifuged at 800 *g* for 5 min, the supernatant was discarded, and the pellet was re-suspended and cultured in D-MEM supplemented with 10% fetal bovine serum (FBS; Invitrogen; 16000), 1% pen-strep (Invitrogen; 15140) and 0.2% L-Glutamine (Invitrogen; 25030) at 37°C in a humidified atmosphere of 5% CO_2_ in air. The medium was changed every third day until confluence when cells were harvested using trypsin 0.05% with EDTA (Invitrogen; 25300), re-suspended in medium and seeded at a 1:3 ratio. Only cells from the third to sixth passage were used for experiments. Experiments were carried out in 1% FBS, if not otherwise stated, to limit the influence and unwanted effects of the serum. The serum-reduced/-starved cells still appeared healthy macroscopically, and the growing rate remained in a pattern of steady increase of the metabolic activity over several days, although the increase was clearly lower than in the 10% FBS conditions.

### Mechanical loading of tendon cells

**Figure 2:**
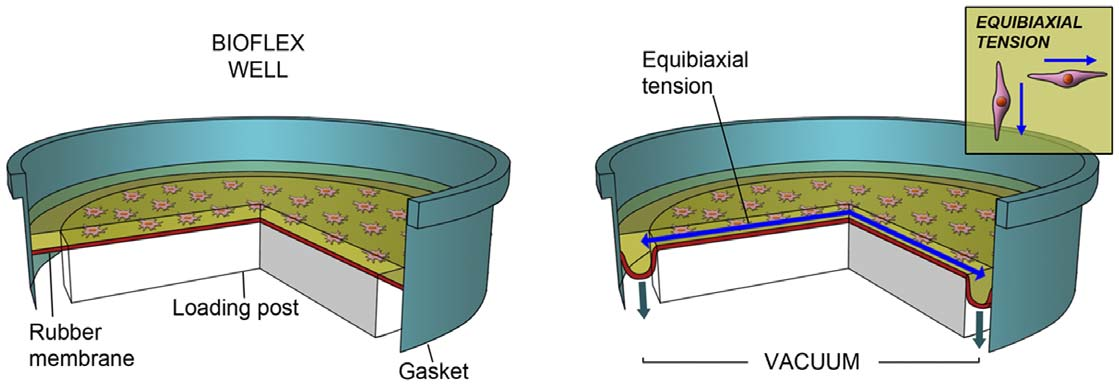
FlexCell© system for 2D-loading of tenocytes. Schematic picture illustrating the function of the FlexCell© system. Vaccum traction applied to the membrane from the under-surface edge of the cylindrical loading post results in an equibiaxially tension on cultures as the membrane is strained downwards. Artwork by G. Andersson.

Cells were seeded two dimensionally on a Bioflex culture plate membrane treated with collagen I (Bioflex; BF-3001C) at a density of 1.75×10^5^cells/well and settled 1 day prior to cyclic strain. Strain was applied equibiaxially to adherent cells via vacuum deformation of the membrane downwards across a 25 mm diameter cylindrical loading post (Fig. 2). 10% strain was applied with a frequency of 1 Hz for a total of 120 min. Cells were exposed to one bout of cyclic strained per day for 3days, before being analysed. The protocol was based on an earlier study [28]. A FlexCell© unit FX-4000 (FlexCell international corporation) was used to apply strain. See Fig. 2 for further details.

### Immunocytochemistry

1.5×10^4^ cells were seeded per well on 8-well chamber slides (BD Falcon 354118) overnight before being processed for vimentin, tenomodulin, SP, and NK-1 R.

A monoclonal mouse anti-vimentin antibody, clone V9, (Dako, Glostrup, Denmark; code: M0725), a goat polyclonal anti-tenomodulin antibody (Santa Cruz, CA, USA; code: sc-49325), a rabbit polyclonal anti-SP antibody (Serotec, Oxford, UK; code: 8450-0004), and a goat polyclonal anti-NK-1 R antibody (Santa Cruz; code: sc-5220) were used at concentrations of 1∶100 on fixed cell cultures. Cells were fixed in 2% paraformaldehyde in 0.1 M phosphate buffer (pH 7.4) for 5 min followed by 4×3 min wash in phosphate buffered saline (PBS). After blocking with rabbit normal serum, donkey normal serum, swine normal serum, and donkey normal serum, for vimentin, tenomodulin, SP, and NK-1 R, respectively, at a concentration of 1∶20 for 15 min, the culture slides were incubated with the primary antibody 60 min at 37°C. After additional washing and blocking, the secondary antibody, [TRITC rabbit anti-mouse (Dako; R0270), FITC donkey anti-goat (Jackson immunoresearch; 705-095-147), TRITC swine anti-rabbit (Dako; R0156) or FITC donkey anti-goat, for vimentin, tenomodulin, SP, and NK-1 R, respectively] was incubated 30 min at 37°C before washing and mounting in Vectashield H-1000 mounting medium for fluorescence (Vector Laboratories, Burlingame, California, USA). For double-stainings the same protocol was repeated before mounting. A Zeiss Axioskop 2 plus microscope equipped with epifluorescence and an Olympus DP70 digital camera was used for analysis.

### Enzyme immunoassay (EIA)

#### 1. Cell lysis

Cell pellets were suspended and incubated on ice for 30 min in lysis buffer [100 Mm Tris-HCI buffer pH 7.0 containing 1 M NaCl, 2% Bovine Serum Albumine (BSA), 4 mM EDTA, 0.2% Triton X-100, 0.02% sodium azide buffer] supplemented with protease inhibitors Pepstatin A (P4265, 0.1 µg/mL), aprotinin (A1153, 5 µg/mL), antipain (A6191, 0.5 µg/mL), benzamidin (B6506, 167 µg/mL) and PMSF (P7626, 5.2 µg/mL), all from Sigma (Sigma Steinheim Germany). Lysates were centrifuged at 13,000 *g* at 4°C for 15 min and the supernatant was retained. Protein determination was done with Protein Assay Dye Reagent Concentrate (Bio-Rad 500-0006).

#### 2. Processing

SP concentration was analysed with a commercially available immunoassay kit (EK-061-05 Phoenix Pharmaceuticals, CA, USA). The assay was performed according to the manufacturer's instructions. Briefly, protein was added to the blocked wells pre-coated with the secondary antibody and thereafter incubated. Afterwards, rehydrated primary antibody and rehydrated biotinylated peptide were added and incubated followed by incubation with SA-HRP solution. For detection, TMB substrate was added and the absorbance was read at 450 nm.

### Western Blot

#### 1. Cell lysis

Cell lysates were obtained after re-suspending cell pellets in lysis buffer [150 mM Sodium chloride, 1% Triton, 0.5% Sodium deoxycholate, 0.1% Sodium Dodecyl Sulphate (SDS), 50 mM Tris, pH 8.0 and protease inhibitor cocktail 1∶200 (all from Sigma-Aldrich)] on ice for 30 min with constant agitation. After centrifugation 5 min at 11,300 *g* in 4°C, to remove cell debris, protein determination was done with Protein Assay Dye Reagent Concentrate (Bio-Rad 500-0006) using Bovine Albumin Serum (BSA, Sigma A9647) as a standard.

#### 2. Process

Samples of similar total protein concentration were boiled in 2x Lammeli sample buffer (Bio-Rad 161-0737) supplemented with 5% beta-mercaptoethanol (Scharlau Me0095) for 5 min at 95°C before loaded onto SDS-PAGE separating gel. The gels were run at 160 V for 60 min then transferred to Polyvinylidene Fluoride Transfer Membrane (PVDF; Santa Cruz; sc-3723) for 1 h at 100 V. The membrane was blocked in TBS-T supplemented with 5% Marvel non-fat milk powder for 60 min on constant agitation in room temperature, and then incubated with primary antibodies in blocking buffer overnight at 4°C. The primary antibody for phosphorylated ERK1/2 (phospho-ERK1/2) detects phosphorylated Thr202 and Tyr204 of ERK1/2, and was used at a concentration of 1∶2000 (Cell signal, Danvers, MA, USA; code: 4370). The primary antibody for NK-1 R was used at concentrations of 1∶4000 (Sigma-Aldrich, Saint Louis, MO, USA; code: s8305). This was followed by 6×5 min of washing in TBS-T before incubation of HRP-conjugated secondary antibody, 1∶2000 (Cell Signal; code: 7074) at room temperature for 60 min. After 6×5 min washes in TBS-T, membranes were treated with chemiluminescent HRP substrate (GE Healthcare; RPN2132) for 5 min prior to visualization on high performance chemiluminescence film (GE Healthcare; 28-9068-38).

Equal loading was confirmed by re-blotting the membrane with beta-actin (Cell Signal; code: 4967) after treatment with Western blot stripping buffer (Thermo Scientific; 21059) for 25 min at 37°C with constant agitation.

### RNA isolation, reverse transcription, and qPCR

Total mRNA was extracted using an RNA extraction kit (Qiagen; 74106) following the manufacture's protocol. Briefly, cells were washed and placed in RLT buffer supplemented with 1% beta-mercaptoethanol and thereafter pipetted into QIAshredders (Qiagen; 79656) and centrifuged. One volume of 70% ethanol, diluted in RNA free water, was added and the product was transferred into RNeasy spin columns. After one wash in RW1 buffer and two washes in RPE buffer, 30 µl of RNAse-free water was added to collect total RNA. Subsequently, RNA concentration was measured using a spectrophotometer Nanodrop ND-1000 (Thermo Fisher Scientific).

The isolated RNA was reverse transcribed into cDNA using a High Capacity cDNA Reverse Transcription kit (Applied Biosystems; 4368813). A total sample volume of 14.2 µl was diluted in a mixture consisting of 2 µl 10x RT buffer, 0.8 µl 25x dNTP, 2 µl 10x RT random primers, and finally 1 µl 20x multiscribe RTase to obtain a total volume of 20 µl. The conversion of RNA to cDNA was done with the following settings; 10 min at 25°C followed by 120 min at 37°C before being paused at 4°C on a thermal cycler (Eppendorf Mastercycler EP Gradient S, Eppendorf, North America).

Quantitative PCR (qPCR) was performed using TaqMan fast universal PCR master mix (Applied Biosystems; 4352042) and probes for TAC1 (Applied Biosystems; code: Hs00243225) and NK-1 R (TACR1; Applied Biosystems; code: Hs00185530). TAC1 is a synonym of preprotachykinin A (PPT-A), i.e. the gene from which the SP precursors derive. For reactions, each sample was run in technical duplicates of a 10 µl from a starting volume of 25 µl consisting of 2 µl cDNA, 12.5 µl master mix, 1.25 µl of 20x probes and 9.25 µl RNase free water. For each amplification, a total of 20 ng cDNA was utilized. The following conditions for amplification were used; after 20 s of denaturation at 95°C the amplification was carried out consisting of 3 s at 95°C for denaturation and 30 s at 60°C for annealing/extension with the 7500 Fast Real-Time PCR systems (Applied Biosystems; version 2.0.1). The expression level of the specific genes was determined relative to that of 18 s RNA, ribosomal RNA control reagents (Applied Biosystems; 4308329).

### Substances for experimental designs

For experimental trials, the cells were incubated in SP peptide (Calbiochem, San Diego, USA; code: 05-23-0600) at a concentration of 10^−7^ M, based on earlier studies [20], [29]. For evaluation of specific receptor mediated effect, an NK-1 R antagonist (Sigma; code: s3144) was used, and found to have optimal blocking effect at a concentration of 10^-6^ M. The NK-1 R antagonist was incubated 30 min prior to the exposure of SP in the blocking experiments.

### MTS assay-cell viability determination

The colorimetric MTS assay was performed to assess metabolic activity in cells. This assay is composed of solutions of a tetrazolium compound [3-(4,5-dimethylthiazol-2-yl)-5-(3-carboxymethoxyphenyl)-2-(4-sulfophenyl)-2H-tetrazolium, inner salt; MTS] and an electron coupling reagent, phenazine methosulfate (PMS), which is bioreduced into formazan by living cells. The resulting formazan precipitate was measured at 490 nm using an enzyme-linked immunosorbent assay (ELISA) reader.

Cells were plated at a density of 5×10^3^ cells per well on a 96-well plate (Corning; Costar 3300) and allowed to adhere and settle overnight followed by serum-starvation 24 h before SP was added for 48 hours, and then MTS was added according to instructions. The metabolic activity is expressed as a value of absorbance in cells and medium normalized to the absorbance of the medium.

### Crystal violet

For determination of viable cell number, crystal violet staining was performed. Cells were cultured at a density of 1.5×10^5^ cells per well on a 6-well plate in triplicates. After allowing the cells to adhere overnight they were serum starved for 24 h, then exposed to SP and the NK-1 R blocker at concentrations stated above, and finally analyzed after an additional 24 h. Non-adherent cells were removed by carefully washing them twice in PBS. The remaining adherent cells were fixed in 1% glutaraldehyde for 30 min. After further washing in PBS, cells were stained for 30 min in 0.1% crystal violet (Sigma; code: C3886) followed by washing in an abundance of water. Cultures were air dried and dissolved in 30% methanol and 10% acetic acid and placed on a shaker for 10 min before the absorbance was read at 590 nm.

### 5-Bromo-2′-deoxy-uridine (BrdU) cell proliferation assay

Cells were plated at a density of 1.5×10^4^ cells on an 8-well chamber slides (BD Falcon; 354118) in triplicates, incubated overnight, then serum-starved for 24 h. After serum-starvation cells were exposed to SP and the NK-1 R blocker at concentrations stated above.

BrdU labelling reagent was added to each well and cells were re-incubated in a humidified atmosphere of 5% CO_2_ for 120 min. Cells were then fixed with ethanol (50 mM glycine, 100 ml EtOH, pH2.0) for 20 min at -20°C and subsequently covered with Anti-BrdU monoclonal antibody, which binds to BrdU incorporated to DNA, at 37°C for 30 min. This was followed by incubation with anti-mouse-Ig-alkaline phosphatase for 30 min at 37°C before cells were covered in freshly made substrate solution (100 mM Tris HCI, 100 mM NaCl, 50 mM MgCl_2_, ph 9.5) supplemented with 4.3 mM NBT and 3.3 mM BCIP. Cells were mounted in Vectashield hard set medium with DAPI (Vector Laboratories H-1500). Results are presented as the percentage of proliferating cells (ratio proliferative cells/all viable cells).

### Statistics

All data were analyzed using PASW Statistics 18 (18.0.0; SPSS Inc., Chicago, IL, USA). Statistical tests used (one-way ANOVA, followed by Bonferroni post-hoc test, and independent samples t-tests) are accounted for in the ‘Results’ section, when the test in question is applied. All results were successfully repeated at least once. Significance was predetermined at p<0.05.

## Results

### Immunocytochemical staining; phenotyping of cells and expression of the SP system

**Figure 3:**
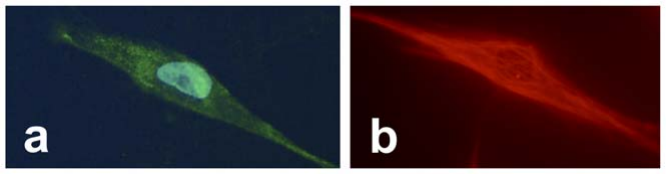
Phenotyping of tenocytes by immunocytochemistry. Two cells from the primary human Achilles tendon cell cultures stained with immunocytochemical methods (FITC in a; TRITC in b). The cell in **a** shows immunoreactions for tenomodulin (green), the cell being counterstained with DAPI to mark the nucleus (bluish), and the cell in **b** is immunostained for vimentin (red).

**Figure 4:**
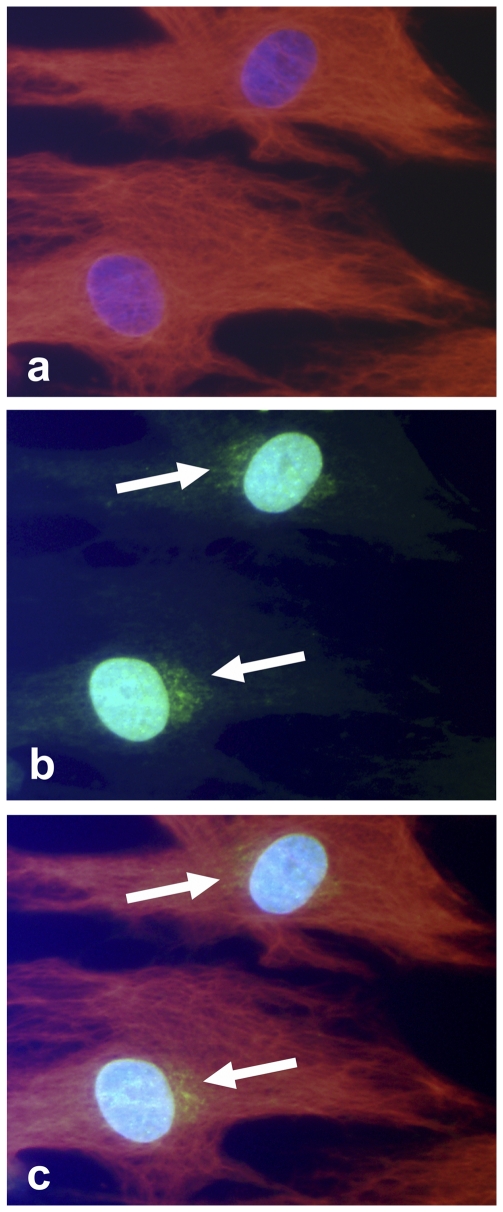
Human Achilles tendon cells in culture immunopositive for SP and vimentin. (**a**) Cells stained with DAPI (bluish), to mark nuclei, and immunolabeled for vimentin (red; TRITC-staining). (**b**) Same cells stained with DAPI and immunolabeled for SP (green; FITC-staining). Clear SP-reactions are seen close to the nuclei (arrows). (**c**) Digitally merged image of *a* and *b*; SP appearing in yellow colour.

The vast majority of cultured tendon cells showed a clear elongated fibroblastic appearance under the microscope (Fig. 3), also evident with regular light microscopy. Very occasionally, a few cells showed a more plump appearance and sporadically some cells exhibited extended dendritic-like processes (cf. below). The majority of the cells were immunopositive for the tenocyte/fibroblast markers tenomodulin (Fig. 3a) and vimentin (Fig. 3b, Fig. 4a).

**Figure 5:**
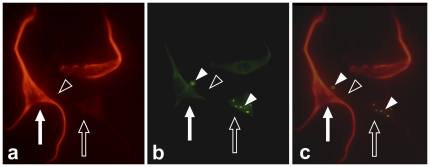
Cells from primary human Achilles tendon cell cultures showing dendritic-like processes. The cells are double-stained for SP (**a**; red; TRITC-staining) and the SP-receptor, NK-1 R (**b**; green; FITC-staining). The pictures (same cells shown in different filters) show that one cell (filled arrow), positive for SP, and another cell (unfilled arrow), negative for SP but displaying positive NK-1 R reactions (filled arrowheads), are making contact with each other via a dendritic-like process (unfilled arrowhead) from the latter cell. Digitally merged image in **c**.

Immunoreactions for SP (Fig. 4b, Fig. 5a) and NK-1 R (Fig. 5b) were regularly observed. When counterstaining tenomodulin-/vimentin-positive cells with SP, the cells were frequently found to express SP-positive intracellular immunoreactions of a punctuate (vesicular) appearance (Fig. 4). Interestingly, a few other SP-positive cells, with pronounced dendritic-like processes, were seen to make interactions with cells positive for the preferred SP receptor, NK-1 R (Fig. 5).

### Detection of SP and NK-1 R proteins confirmed by EIA and Western blot

In addition to immunocytochemical findings, the presence of SP protein in primary tendon cell cultures was confirmed with EIA. The mean concentration of SP was 170 pg/2×10^6^ tendon cells.

**Figure 6:**
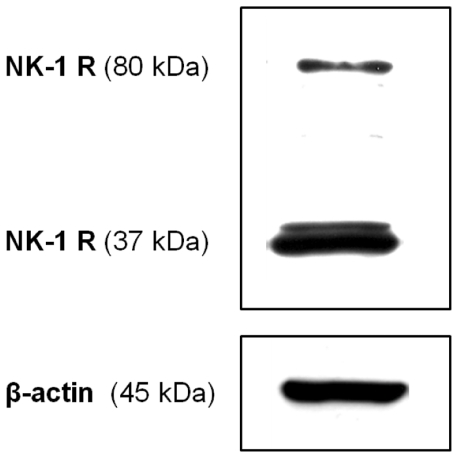
NK-1 R in human primary Achilles tendon cells. Two clear bands of 80 kDa and 37 kDa, respectively, are shown. β-actin is shown as a reference.

Western blot confirmed the presence of NK-1 R in the cultured tendon cells (Fig. 6). Two clear bands (80 kDa and 37 kDa) were present as expected; these bands are likely to represent the full-length glycosylated and the truncated unglycosylated forms of the receptor respectively.

### SP mRNA increases and NK-1 R mRNA decreases in response to mechanical loading

**Figure 7:**
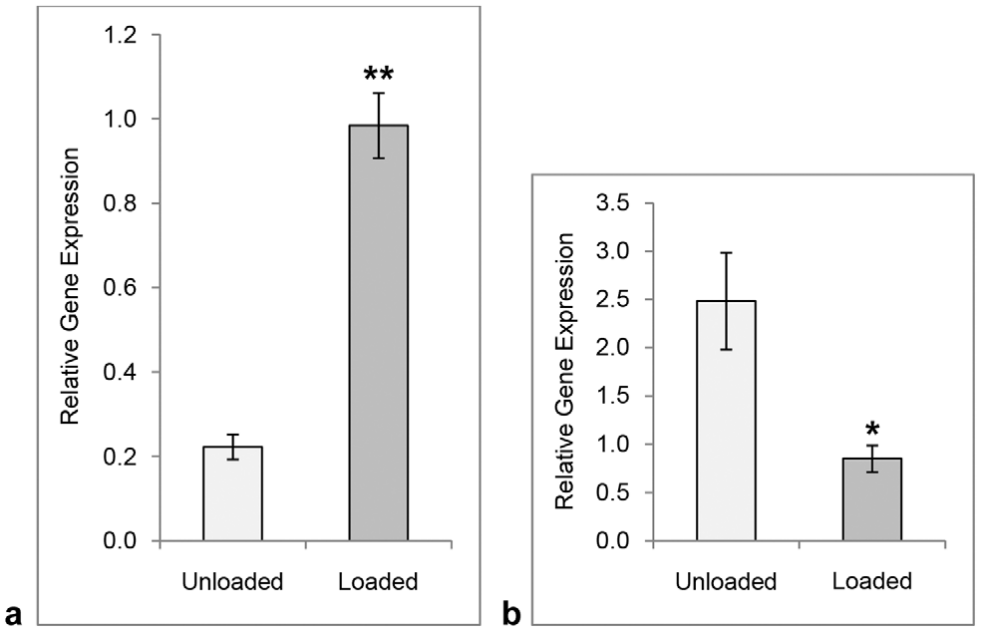
Results of loading on gene expression. qPCR analysis for SP (**a**) and NK-1 R (**b**) mRNA, respectively, in cultured human primary Achilles tendon cells. The mean level of SP mRNA is significantly elevated after 2D-loading of the cultures, whereas the mean level of NK-1 R mRNA is significantly lowered after the loading. Error bars represent standard error of the mean. Level of significance: * p<0.05; **p<0.01 (Independent samples t-test).

The mean level of SP mRNA, as measured by qPCR with a primer-probe set targeted against TAC1, was significantly higher (>4 fold increase) in cell cultures that had been submitted to the mechanical loading protocol compared with cell cultures that had not (Fig. 7a; Independent samples t-test). The opposite result was noted for the SP receptor, i.e. NK-1 R mRNA was significantly lower in loaded cell cultures than unloaded (Fig. 7b; Independent samples t-test).

### NK-1 R expression decreases after submission of SP

**Figure 8:**
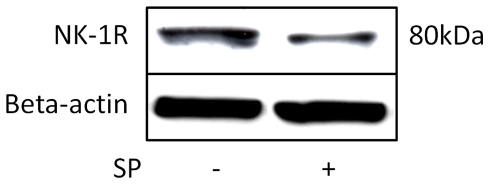
Effect of SP incubation on NK-1 R expression. Full-length glycosylated NK-1 R expression (Western blot) in cultured human primary tendon cells is lower in cells incubated with SP (10^−7^ M) for 4 hours (right) as compared to control cells (left).

The expression of the full-length glycosylated NK-1 R, as seen with Western blot, was lower in cells incubated with SP for 4 hours as compared to control cells (Fig. 8).

### SP stimulates tendon cell metabolic activity and proliferation, and activation of ERK1/2

**Figure 9:**
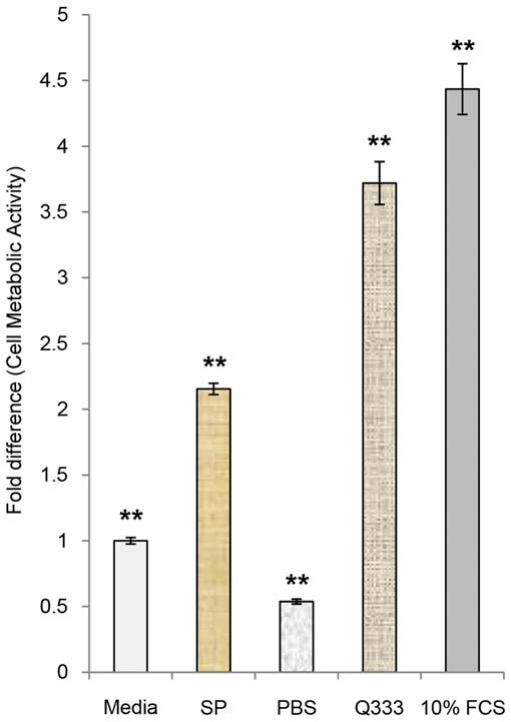
Metabolic activity after SP incubation. Incubating human tendon cells with SP (10^−7^ M) for 48 hours significantly increases their metabolic activity compared to incubation with pure cell culture media or PBS. The potency of SP as a proliferative factor is about half that of cell culture media with 10% fetal calf serum (FCS). As another positive reference, results from incubation with a cell culture medium with a serum-free growth factor (Q333) are shown. Results from MTS-assay analysis. Serum-free media set as standard to 1. Error bars represent standard deviation. **p<0.01.

Initial results of MTS-assay 48 hours after adding SP to cells in serum free culture media, showed that SP significantly increased the metabolic activity in the cultures, as compared to incubation with pure media or ordinary PBS, but not as much as incubation with cell culture media with 10% fetal calf serum or a serum-free growth factor (Q333; PAA; U15-813) (Fig. 9; One-way ANOVA, followed by Bonferroni post-hoc test.).

**Figure 10:**
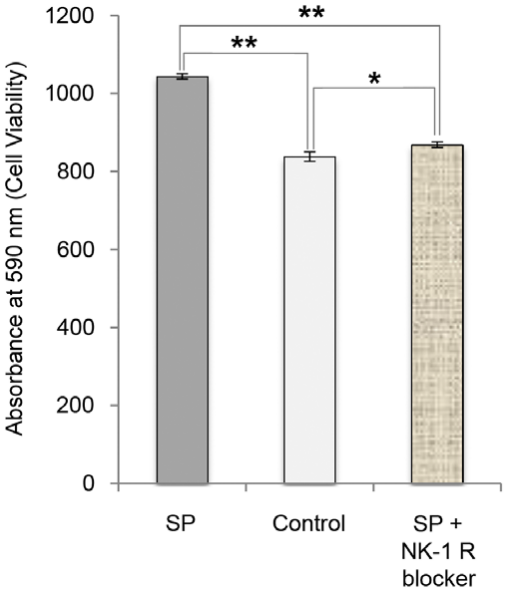
Cell viability after SP incubation and NK-1 R blocking. Analysis of viable tendon cells in human primary cultures after 24 hours of incubation with SP (10^−7^ M), without SP (control), and with SP and the NK-1 R blocker (10^−6^ M), as measured with crystal violet staining. The significant increase in viable cells seen after incubation with SP is effectively blocked with the NK-1 R antagonist. Error bars indicate standard deviation. *p<0.05; **p<0.01.

The effects of SP on the number of viable tendon cells was confirmed with another method (crystal violet staining; Fig. 10), and the significant increase in the number of cells seen after 24 hours of incubation with SP was almost entirely blocked with the NK-1 R antagonist (Fig. 10; One-way ANOVA, followed by Bonferroni post-hoc test).

**Figure 11:**
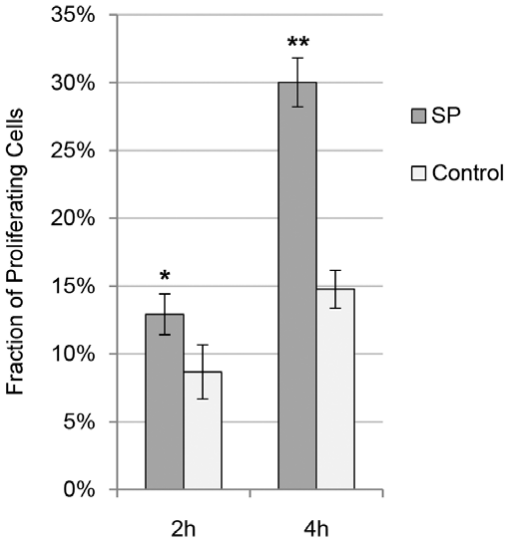
SP effect on cell proliferation at different time-points. The mean fraction of proliferating (BrdU-positive) tendon cells in human primary cultures after incubation with SP (10^−7^ M) was seen to be significantly higher than in cultures not incubated with SP (control) at both 2 and 4 hours of incubation. Error bars represent standard deviation. *<0.05; **p<0.01 (Independent samples t-test).

**Figure 12:**
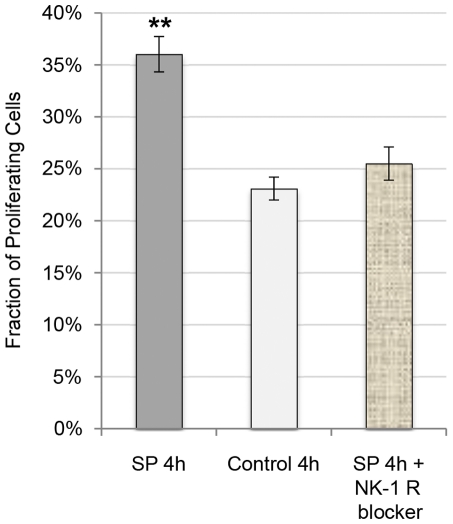
Cell proliferation after SP incubation and NK-1 R blocking. The mean fraction of proliferating (BrdU-positive) tendon cells in human primary cultures after 4 hours of incubation with SP (10^−7^ M), without SP (control), and with SP and the NK-1 R blocker (10^−6^ M). The significant increase in the percentage of proliferating cells seen after incubation with SP is effectively abolished with the NK-1 R blocker. Error bars represent standard deviation. **p<0.01.

When measuring the percentage of proliferating (BrdU-postive) tendon cells in cultures after incubation with SP, the fraction of proliferating cells was significantly increased after both 2 and 4 hours of SP incubation as compared to controls (Fig. 11). This effect of SP was blocked with the NK-1 R antagonist (Fig. 12; One-way ANOVA, followed by Bonferroni post-hoc test).

**Figure 13:**
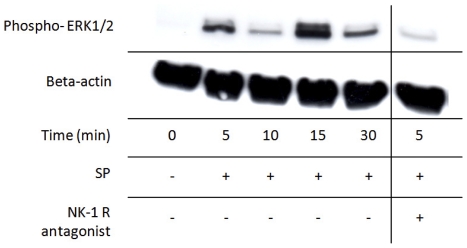
SP effect on activation of mitogen-activated protein kinases. Phosphorylated ERK1/2 in cultured human Achilles tendon cells at different time points after incubation with SP (10^−7^ M). The results clearly show that SP activates the phosphorylation of ERK1/2 over time in a biphasic course that peaks at 5 and 15 minutes of exposure. The activation by SP is effectively blocked when incubated simultaneously with the NK-1 R blocker (10^−6^ M; result at 5 min to the right). Beta(β)-actin is shown as a reference.

Complementary Western blot analysis showed that the exogenously administered SP stimulated phosphorylation of ERK1/2 in the cultured cells; this activation displayed a biphasic course, peaking after 5 and 15 minutes of exposure (Fig. 13). The ERK1/2 activation by SP was effectively blocked when incubated simultaneously with the NK-1 R blocker (Fig. 13).

## Discussion

### Primary human Achilles tendon cell culture model

This study establishes primary human Achilles tendons cells as a valid model for studying effects of neurochemical mediators, in this case SP, on human tenocytes. Studies on tendon pathology using cell cultures are common, although most research is performed on animal-derived tendon cells and/or established/immortalised cell lines. Nevertheless, other groups have successfully grown primary human Achilles tendon cells in culture (e.g., [30], [31], [32]). In our study, the overwhelming majority of cultured cells showed characteristics of a fibroblastic phenotype, retained in passages used for experiments (three through six), confirming that the study is performed on cell cultures consisting almost entirely of tenocytes (i.e. fibroblasts derived from the tendon proper). However, one should keep in mind that tendons *in vivo* also consist of other cell types than tenocytes, such as chondrocytes and endothelial cells [33], adipocytes [34], and nerve cells [5]. It is also known that the tendons harbour tendon stem cells that can differentiate into adipocytes, chondrocytes and osteocytes [35]. A few cells of the primary cultures of the present study actually did display rare positive reactions of such cell types (data not shown). Regarding nerve cells, it is also observed that some cells in the cultures of this study have dendritic-like processes, which further indicates that nerve cells might be present.

To ensure that the majority of our cells were tenocytes, we phenotyped the cell population with vimentin, a type III intermediate filament seen in mesechymal derived cells such as fibroblasts [36], and also tenomodulin, a type II transmembrane glycoprotein which is predominantly expressed in tendon and ligaments, and is considered to be a marker fairly specific for tenocytes [37], [38], [39].

### Human Achilles tendon cells produce SP and express NK-1 R in culture

This study produces direct evidence that human tendon cells produce SP as shown by immunocytochemistry and EIA. Previously, only SP mRNA has been detected in human tenocytes [11]. The concentration of SP detected (170 pg/2×10^6^ tendon cells) seems reasonable; rat thymocytes for instance produce SP in the range of 50–175 pg/5×10^6^ cells [40]. It should be noted, that in a rat tendinosis model (flexor digitorum tendons), SP-immunoreactivity was shown in additional cell types associated with tendons, aside from tenocytes, such as peritendon mast cells and macrophages [41].

Furthermore, it is clear that the tendon cells still express NK-1 R *in vitro*. This has previously been shown in studies on human tendon tissue sections [11], [17]. This is important to establish, since it provides a theoretical basis for specific SP effects to be exerted on the cultured cells, as in *in vivo* situations. The NK-1 R detection via Western blot clearly showed two bands (80 kDa and 37 kDa), which likely represent the full-length glycosylated and the truncated unglycosylated form of the receptor, respectively. It is known that both types of the receptor have similar affinity to SP. However, regarding phosphorylation of ERK1/2, it is previously shown that stimulation of the full-length glycoslated form of NK-1 R more rapidly results in ERK1/2 phosphorylation [42], [43]. This most likely explains the biphasic peaking of ERK1/2 phosphorylation in our result, the full-length glycosylated receptor peaking at 5 min and the truncated unglycosylated receptor peaking at 15 min.

An interesting finding was that the Achilles tendon cells appeared to form functional interactions in the cultures, SP-positive cells making close contact with NK-1 R-positive cells.

### SP production increases with mechanical loading whereas NK-1 R expression decreases

When subjecting two-dimensionally cultured tenocytes to cyclic load according to a protocol (10% strain, 1 Hz, 120 min every day for 3 days), SP mRNA was significantly increased (more than four-fold) as compared to unloaded control cells. This is in agreement with our *in vivo* model of tendinosis, in which we recently demonstrated that a repetitive loading protocol of the triceps surae muscle, with periods of rest in between, accelerates intratendinous SP production in the Achilles tendon tissue of rabbit [26]. In humans, circulating levels of SP have been shown to be elevated after exercise [44]. Regarding the loading protocol used here, in a recent study on tenocyte differentiation in cells from a mesenchymal cell line, we showed that loading of the cells in a 3D collagen environment (bioartificial tendons) most efficiently increased tenocyte gene expression when cyclic loading was applied, and periods of rest inserted between loading further enhanced tenocyte gene expression as did increasing strain levels up to 10% [28]. We did not assess the affect of three-dimensional vs two-dimensional culturing in the current study, but this may be worthy of further research in the future. Nonetheless, in this case the 2D *in vitro* system performed well as a way to model the effects already observed *in vivo*.

Contrary to the results of SP, the present study shows that NK-1 R mRNA in tendon cells is significantly decreased (about three times), when cells are submitted to mechanical loading. One possible explanation for this is that the NK-1 R decrease is secondary to the SP increase, this being the result of a negative loop, i.e. the more SP that stimulates NK-1 R, the fewer receptors are expressed [45]. Indeed, this theory seems to be strengthened by additional results of the present study, showing that Western blot analysis of NK-1 R expression is lower in the tendon cells after 4 hours of incubation in 10^−7^ M of SP.

It has also been shown that exposure of SP on hamster ovary cells causes a decrease of 90% in NK-1 R responsiveness, desensitization, along with a decrease of 50% in the amount of membrane binding sites as the receptor internalizes following binding to SP [46]. However, during resensitization, there is a significant increase in receptor responsiveness without the membrane binding sites being increased, i.e. before the receptor is recycled [46].

We have also found a similar response (divergent mRNA levels for SP and NK-1 R) in human (hamstring) tenocytes when co-cultured with mast cells, i.e. SP mRNA is decreased when tenocytes are co-cultured whereas NK-1 R mRNA is increased. Thus it appears that coordinate regulation of SP and NK-1 R may be a general phenomenon in human tenocytes (unpublished data).

### Tenocyte proliferation and metabolic activity is increased after exogenous SP stimulation; specific NK-1 R mediated effects possibly via activation of mitogen-activated protein kinases

This study shows that when primary human tenocytes in culture are submitted to exogenous SP, this activates the phosphorylation of the mitogen-activated protein kinases ERK1/2 already after 5 minutes. This activation showed a biphasic course, peaking at 5 and 15 minutes, which might correspond to the different forms of the receptor [42], [43]; the activation via the full-length glycosylated receptor peaking at the earlier time point and the activation via the truncated unglycosylated receptor peaking at the later time point. The phosphorylation of ERK1/2 by SP is effectively blocked by an NK-1 R inhibitor, confirming that the SP effect is specifically mediated primarily via this receptor. It is well known that GPCRs like NK-1 R are involved in mitogenic signals leading to proliferation; activation via mitogen-activated protein kinases being one such well-known pathway [19]. In colonocytes it has been shown that SP increases cell proliferation via ERK1/2 through an epidermal growth factor receptor (EGFR) signalling pathway [20].

After an additional 2–4 hours of incubation with SP, it is here shown that the fraction of proliferating human tenocytes increases significantly (between 1.6–2 fold increase after 4 h), and that this SP effect is also blocked by the NK-1 R inhibitor. One to two days (24–48 h) after SP exposure the number of viable cells and the metabolic activity were significantly increased, strengthening that SP indeed stimulates proliferation of human tenocytes; this being specifically mediated via NK-1 R and possibly through the activation of ERK1/2.

These results are again in accordance with the findings of our studies in the *in vivo* tendinosis model. The increase in the load-induced endogenous SP production seen in the rabbit Achilles tendon was found to precede the tendinosis tissue changes, including tenocyte hypercellularity [26], and exogenously administrated SP accelerated the tendon cell proliferation [27].

Earlier studies on the effects of SP on human fibroblasts (skin derived) in culture, have also shown that SP, under certain conditions can exert proliferative effects on these cells [47], [48], and paratendinous injections of SP also enhances cell proliferation in rat Achilles tendons during wound healing [13], [14]. Most interestingly, exogenously administered SP seems to have a booster effect on endogenous SP for rat fibroblast proliferation via autocrine/paracrine stimulation in an Achilles tendon rupture model [49].

### Conclusions

In summary, this study confirms that human Achilles tendon tenocytes are capable of producing SP and that they express NK-1 R. The results furthermore suggest that when tenocytes are submitted to mechanical loading, the SP production increases, providing a possible explanation of elevated peripheral SP levels in strain-induced conditions, such as tendinosis [26]. Moreover, it is here experimentally shown that SP triggers proliferation of human Achilles tendon tenocytes through a NK-1 R specific pathway, giving further evidence of a possible involvement of SP in the pathology of tendinosis, specifically in the excessive cell proliferation that occurs [1]. The activation of MAPKs by SP is in accordance with established knowledge on GPCR mediated proliferative effects; further validating the importance of the NK-1 R.

In conclusion, the present study indicates that SP, produced by tenocytes in response to mechanical strain, regulates cell proliferation through an autocrine loop involving the NK-1 R.
